# Patient experiences of GP-led colon cancer survivorship care: a Dutch mixed-methods evaluation

**DOI:** 10.3399/BJGP.2022.0104

**Published:** 2022-11-01

**Authors:** Julien AM Vos, Vera E van Miltenburg, Frédérique H Beverdam, Henk CPM van Weert, Kristel M van Asselt

**Affiliations:** Department of General Practice, Amsterdam University Medical Centers, University of Amsterdam; Programme for Personalised Medicine, Amsterdam Public Health Research Institute, Amsterdam, the Netherlands.; Department of General Practice, Amsterdam University Medical Centers, University of Amsterdam, Amsterdam, the Netherlands.; Department of Surgery, Schiedam, the Netherlands.; Department of General Practice, Amsterdam University Medical Centers, University of Amsterdam; Programme for Personalised Medicine, Amsterdam Public Health Research Institute, Amsterdam, the Netherlands.; Department of General Practice, Amsterdam University Medical Centers, University of Amsterdam; Programme for Personalised Medicine, Amsterdam Public Health Research Institute, Amsterdam, the Netherlands.

**Keywords:** aftercare, colon cancer, patient satisfaction, primary health care, quality of health care, survivorship

## Abstract

**Background:**

Colon cancer survivorship care constitutes both follow-up and aftercare. GP involvement may help to personalise care.

**Aim:**

To explore patients’ experiences of GP-led versus surgeon-led survivorship care.

**Design and setting:**

Patients with stage I to III colon cancer were recruited from eight Dutch hospitals and randomised to receive care by either the GP or surgeon.

**Method:**

A mixed-methods approach was used to compare GP-led care with surgeon-led care. After 1 year the Consumer Quality Index (CQI) was used to measure quality aspects of care. Next, interviews were performed at various time points (3–6 years after surgery) to explore patients’ experiences in depth.

**Results:**

A total of 261 questionnaires were returned by patients and 25 semi-structured interviews were included in the study. Overall, patients were satisfied with both GP-led and surgeon-led care (ratings 9.6 [standard deviation {SD} 1.1] versus 9.4 [SD 1.1] out of 10). No important differences were seen in quality of care as measured by CQI. Interviews revealed that patients often had little expectation of care from either healthcare professional. They described follow-up consultations as short, medically oriented, and centred around discussing follow-up test results. Patients also reported few symptoms. Care for patients in the GP-led group was organised in different ways, ranging from solely on patient’s initiative to shared care. Patients sometimes desired a more guiding role from their GP, whereas others preferred to be proactive themselves.

**Conclusion:**

Patients experienced a high quality of colon cancer survivorship care from both GPs and surgeons. If the GP is going to be more involved, patients require a clear understanding of roles and responsibilities.

## INTRODUCTION

Cancer survivorship care is characterised by its broad and multidimensional approach. It is not only limited to the detection of possible recurrences, but also constitutes aftercare, such as psychological and social support, attention to rehabilitation, reintegration to society, and secondary prevention.^1^ In most Western countries, survivorship care for patients with colon cancer is provided by a medical specialist. During routine follow-up consultations in the hospital, patients express many different needs.^2^^,^^3^ Due to the growing number of cancer survivors who need to be monitored over time, the demand for other and more personalised strategies is increasing.^4^^,^^5^ Elements of general practice care could be of additional value.^6^^,^^7^ Earlier studies on GP-led versus specialist-led survivorship care show similar clinical and patient-reported outcomes, while also resulting in lower healthcare costs.^8^^,^^9^ The Dutch Ministry of Health, Welfare and Sports has therefore advocated a greater role for the GP after cancer treatment.^10^ These considerations led to the initiation of the ‘Improving care after colon cancer treatment in the Netherlands; personalised care to enhance quality of life’ (I CARE) study in 2015, comparing GP-led with surgeon-led survivorship care for patients with colon cancer, with or without access to an eHealth application (Oncokompas).^11^ Within the first year after surgery, involvement of the GP did not improve or decrease quality of life (QoL) recovery, the primary outcome of the study.^12^

Besides investigating patient self-reported outcome measures, such as health-related QoL, there has been a growing interest in patient-reported experience measures providing insight into the patients’ experiences with care.^13^^,^^14^ These experiences help to evaluate new models of survivorship care and have been regarded as quality indicators of patient care and safety. In this mixed-methods study, the researchers aimed to explore patients’ experiences with survivorship care provision by the GP versus by the surgeon, using both patient questionnaire data and in-depth semi-structured interviews. It was hypothesised that GP involvement, and presumably its increased attention to aftercare and rehabilitation, would result in improved patient satisfaction and improved cancer care experiences.

## METHOD

### I CARE study

The I CARE study is a multi-centre 2 × 2 factorial randomised controlled trial comparing GP-led with surgeon-led (usual) survivorship care for limited-stage (I, II, and III) patients with colon carcinoma in which Eight Dutch hospitals participated. Patients were also randomised for access to ‘Oncokompas’, a supporting eHealth application that aims to increase patient knowledge on cancer and facilitate access to supportive care. The primary outcome was QoL. A full description of the study procedures and recruitment period have been previously published.^11^^,^^15^ Box 1 provides the definition of colon cancer survivorship care and how it was operationalised for this study. Questionnaires were sent out to patients after 3, 6, 12, and up to 60 months of follow-up.

**Box 1.** I CARE intervention

Survivorship care involves both follow-up and aftercare. It can be defined according to the recommendations of the Institute of Medicine (IOM):^1^ prevention of recurrent and new cancers, and of other late effects; surveillance for cancer spread, recurrence, or second cancers; assessment of medical and psychosocial late effects; intervention for consequences of cancer and its treatment; and coordination between specialists and primary care providers to ensure that all of the survivor’s health needs are met. All of these aspects of survivorship care are incorporated in the Dutch national follow-up guideline for colorectal cancer.^19^ For the I CARE study, the follow-up guideline was summarised in a survival care plan and provided to the participating GPs before the start of the intervention. The survival care plan did not contain any personalised information or recommendations for the patient, but included general information on follow-up schedule, management of symptoms, and treatment side effects. The patient and GP were free to organise care as they deemed fit. Patients were allowed to transfer from the GP back to the surgeon at any point in time.

**Table:** How this fits in

Cancer survivorship care is often complex and requires a multidimensional approach. This study found that patients receiving colon cancer survivorship care from either the GP or surgeon rated the received care as of high quality. In the future, roles and responsibilities of patients and physicians need to be clear in order to help organise survivorship care. GPs can take on a more prominent role in cancer survivorship care, but other outcomes, including patients’ and physicians’ preferences, will also be important.

### Quantitative data collection and analysis

This mixed-methods study addressed a secondary outcome of the I CARE study, namely patient experiences and satisfaction with care. The standardised Consumer Quality Index (CQI) was used to measure patient experiences.^16^ The CQI for ‘General practice care’ was adapted to fit the purpose of the intervention and enable the comparison between GP- and surgeon-led care.^17^ The adapted CQI included quality domains relating to communication and information; comprehensiveness of care, for example, attention to psychosocial and emotional problems, and preventive care; and attitude of the healthcare provider. The domains consisted of five to eight items per domain. Finally, the CQI included single questions on the accessibility of care (two items), whether or not the patient would recommend the outpatient clinic or general practice to their friends and family (one item), and patients’ satisfaction (three items). An open-ended question was posed to enquire about suggested care improvements (an English translation of the adapted CQI can be found in Supplementary Appendix S1).

After 1 year of follow-up CQI data were analysed, in which period the greatest differences were expected. Scoring was according to the CQI user manual, in which higher scores indicate better experiences with care.^18^ To assess the internal consistency of the CQI domains, reliability analyses were carried out using Cronbach’s alpha and inter-item correlations, in which an α≥0.7 and *r*≥0.3 were considered acceptable. Summed mean scores and standard deviations (SDs) for domains and single items were calculated. Independent sample *t*-tests were performed to test the differences between groups. A two-sides *P*>0.05 was considered statistically significant. All analyses were performed according to the intention-to-treat principle, using IBM SPSS Statistics (version 26).

### Qualitative data collection and analysis

To gain a comprehensive insight into patient experiences, patients from both the GP-led and surgeon-led trial arms were interviewed at various time points in their follow-up programme (range 3–6 years after surgery). All patients had finished their 3-year follow-up period, during which most follow-up consultations take place according to the national guideline.^19^ In total, 26 interviews were held. Participation was on a voluntary basis. A call was placed in the yearly I CARE study newsletter, after which 10 patients were included.

Purposive sampling was used next to obtain a representative patient sample. The researchers aimed to include patients who had a (temporary) stoma, and patients who had transferred from the GP back to the surgeon because their experiences with care could differ. Overall 36 patients were contacted via email and telephone, of which 16 patients agreed to participate (eight could not be reached, eight had no interest in an interview, and four mentioned they had little contact with their healthcare provider). Interviewers and patients had no prior relationship.

An interview guide was designed, consisting of two components: starting with an open, narrative component, encouraging patients to elaborate on their experiences, followed by a semi-structured component based on the Quality of Cancer Survivorship Care framework, as proposed by Nekhlyudov *et al* and Kline *et al*.^20^^,^^21^ This is a consensus and evidence-based framework, consisting of nine domains that can be used to evaluate cancer survivorship care. Interview questions were derived from these domains and discussed within the research group. Finally, patients who had randomised to Oncokompas were asked if they had used the application and whether it had been beneficial. The interview guide was pilot tested with four patients, after which minor adjustments were made to optimise and finalise the guide.

The interviews were conducted by the second author and another Master’s student (who is acknowledged). The first four interviews were attended by the first author to guarantee the quality of the interview techniques. All interviews (except one) were held through videoconferencing or telephone due to the security measures surrounding the COVID-19 pandemic. Interviews were audio-recorded, transcribed verbatim, and anonymised by the second author and Master’s student previously mentioned. Transcripts were verified against original audio data by the first author. Mean interview duration was 43 min (range 15–79 min).

Transcripts were analysed using thematic analysis.^22^ Transcripts from the GP-led and surgeon-led care groups were coded and analysed separately. Two independent researchers coded the transcripts (the first author coded all transcripts, the second coded the GP-led, and the same Master’s student previously mentioned coded the surgeon-led group). Interviews were held until no new themes were identified and data were considered rich and sufficient.^23^ Data from the GP-led group were more heterogeneous, which resulted in more interviews to reach data sufficiency. Using an iterative process and frequent discussions within the research group, three key themes were identified. The main findings within these three key themes are described and presented in this article. As part of a member check, a Dutch synopsis of the findings was sent to all participants by email, together with their interview transcript. Of these participants, 10 opened the files, but there were no comments or remarks.

Transcripts were coded using MAXQDA Plus software (version 20.3.0). The Consolidated Criteria for Reporting Qualitative Studies (COREQ) were used for the reporting of the qualitative study results (Supplementary Appendix S2).^24^

## RESULTS

**Figure 1.**
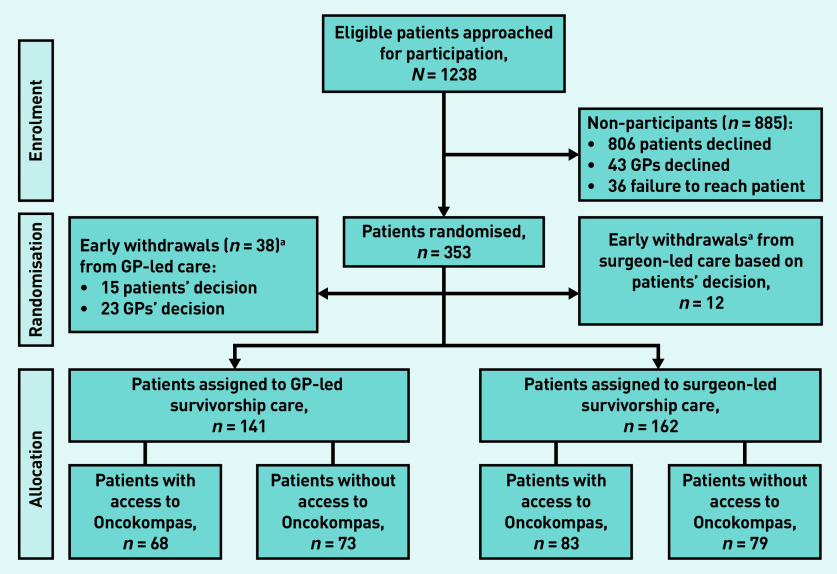
*Trial flow diagram.* *^a^ Participants who withdrew study consent shortly after randomisation.*

Between 26 March 2015 and 21 November 2018 a total of 1238 eligible patients with colon cancer were approached for participation (Figure 1). Of these, 353 patients were randomised, but 50 patients dropped out shortly after randomisation and before the start of the intervention due to the patients’ ( *n* = 27) or GPs’ withdrawal of consent ( *n* = 23). As a result, 303 of 1238 patients (24%) were included. Of these, 141 patients were randomised to the GP-led trial arm (of which 68 had access to Oncokompas) and 162 patients to the surgeon-led trial arm (of which 83 had access Oncokompas). A total of 250 GPs participated in the trial, of which 126 were allocated to provide survivorship care. Table 1 shows the baseline characteristics of all participants. In short, the patient population had a mean age of 68.0 years (SD 8.4) and included more males (67%; *n* = 203). Of the participants 22% ( *n* = 68) received adjuvant chemotherapy. Only 4% ( *n* = 13) of patients had a stoma in the study.

**Table 1.** Baseline characteristics of participants

**Characteristic**	**All participants (*n*= 303)**		**Interview participants (*n* = 25)**	
	
	**GP–led (*n*= 141)**	**Surgeon-led (*n*= 162)**	**GP-led (*n*= 17)^a^**	**Surgeon-led (*n*= 8)**
**Sociodemographic**				
Age, years, mean (SD)	67.9 (8.3)	68.2 (8.4)	67.1 (8.4)	59.5 (6.7)
Sex, male, *n* (%)	98 (70)	105 (65)	14 (82)	4 (50)
Living situation, with a partner, *n* (%)	107 (76)	120 (74)	16 (94)	8 (100)
Educational attainment, *n* (%)				
Primary or none	14 (10)	13 (8)	1 (6)	NA
Secondary	28 (20)	40 (25)	2 (12)	2 (25)
Vocational education	75 (53)	71 (44)	10 (59)	4 (50)
University	12 (9)	24 (15)	3 (18)	2 (25)
Missing	12 (9)	14 (9)	1 (6)	NA
Randomised to Oncokompas, *n* (%)	68 (48)	83 (51)	10 (59)	3 (38)

**Clinical and pathological**				
Comorbidities, *n* (%)				
0–1	63 (45)	84 (52)	12 (71)	5 (63)
≥2	78 (55)	78 (48)	5 (29)	3 (38)
Cancer diagnosis via, *n* (%)				
Population screening	74 (52)	78 (48)	10 (59)	4 (50)
Clinical course	67 (48)	84 (52)	7 (41)	4 (50)
Tumour stage, *n* (%)				
I	59 (42)	54 (33)	4 (24)	3 (38)
II	50 (35)	54 (33)	5 (29)	2 (25)
III	32 (23)	54 (33)	8 (47)	3 (38)
Stoma, *n* (%)	6 (4)	7 (4)	3 (18)	NA
Chemotherapy, *n* (%)	27 (19)	41 (25)	7 (41)	3 (38)
Time since surgery at moment of	NA	NA	4.3 (3.6–5.1)	5.0 (4.3–5.8)
interview, years, median (IQR)				

a *During follow-up six patients transferred from the GP back to the surgeon. IQR = interquartile range. NA = not applicable.*

### Quantitative data

**Figure 2.**
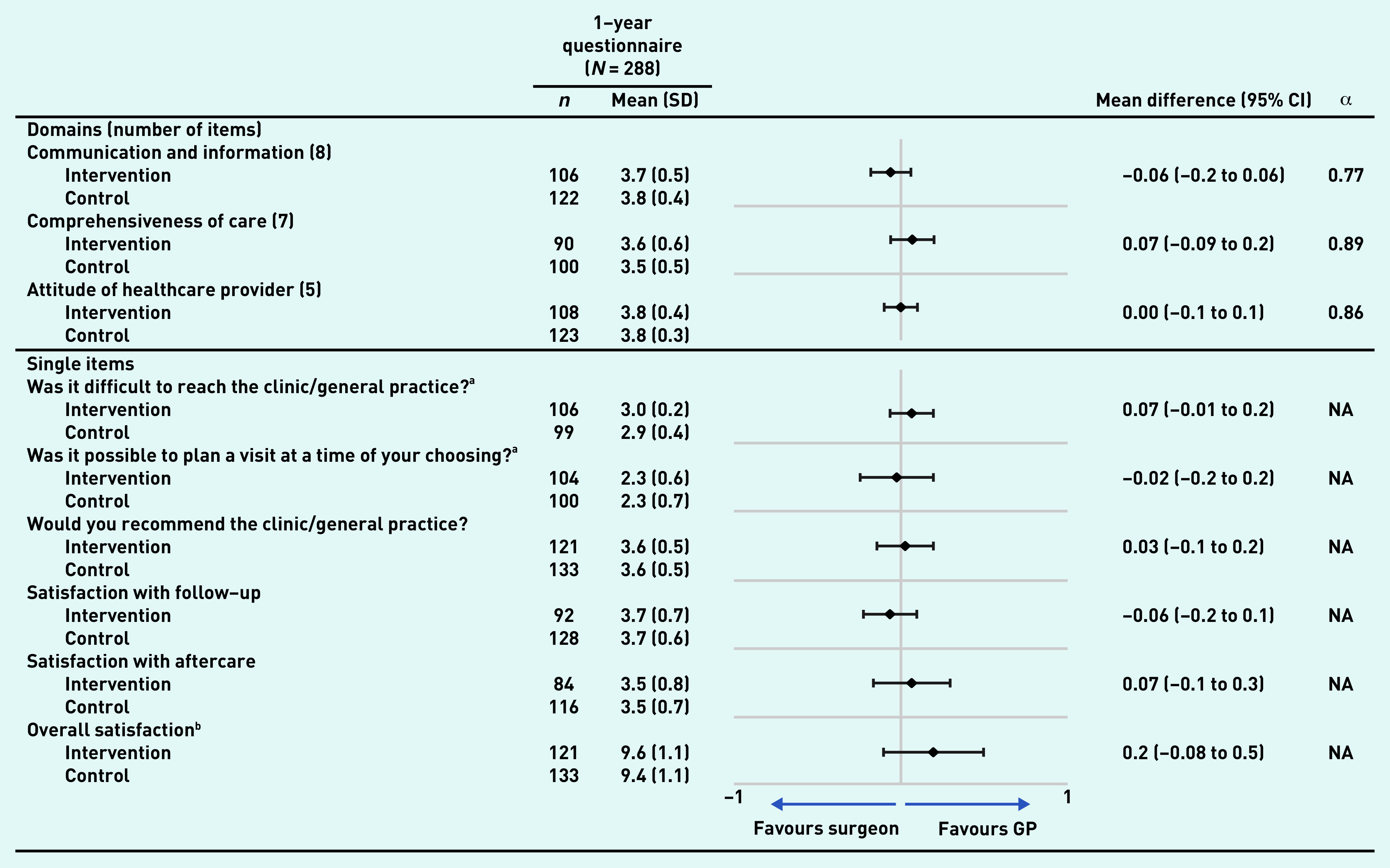
*Quality aspects of care according to the customised Consumer Quality Index. Graph shows the mean differences (and 95% confidence intervals) in ratings of colon cancer survivorship care by a GP versus a surgeon. Values may not total 261 (number of responses received) because of missing data. ^a^ Values range from 1 (very difficult) to 3 (not at all difficult). ^b^Values range from 0 (bad) to 10 (excellent). All other values range from 1 (lowest) to 4 (highest). CI = confidence interval. NA = not applicable. SD = standard deviation.*

After 1 year of follow-up, 288 questionnaires were sent out by email or post (the remaining 15 questionnaires were either suspended at the request of the patients or in one case because of death). Of the questionnaires, 91% ( *n* = 261) were returned. Patients were satisfied with both GP-led and surgeon-led colon cancer survivorship care (Figure 2). Overall satisfaction was 9.6 (SD 1.1) out of 10 with GP-led care versus 9.4 (SD 1.1) with surgeon-led care, resulting in a mean difference of 0.2 (95% confidence interval [CI] = −0.08 to 0.5). No important differences were seen in any quality aspects of care as measured by the CQI. The open-ended question yielded some suggestions regarding care improvement: some patients experienced difficulties with planning and wished for less waiting time ( *n* = 21) (data not shown). Others wished for longer consultation time and increased attention from their physician ( *n* = 13) (data not shown). These suggestions were mentioned in both trial arms.

### Qualitative data

In total, 26 semi-structured interviews were held, of which *n* = 17 patients had been randomised to the GP, and *n* = 9 patients to the surgeon (Table 1). Because of a logistical problem, one interview from the surgeon-led trial arm was not audio-recorded, and therefore not included. Most patients had (nearly) finished the full 5-year follow-up programme (median time since surgery was 4.3 years (interquartile range [IQR] 3.6–5.1) in the GP-led trial arm versus 5.0 years (IQR 4.3–5.8) in the surgeon-led trial arm). During the follow-up six patients transferred from the GP back to the surgeon owing to (suspected) disease recurrence ( *n* = 3), patients’ preference ( *n* = 2), or stoma complication ( *n* = 1).

The following key themes were identified:

- expectations of care;
- experiences with care; and
- roles and responsibilities.

### Expectations of care

Patients’ experiences and satisfaction were influenced by their expectations of care. Motivations to participate in this study differed: some of the incentives were contributing to scientific research, helping other patients with colon cancer, and showing gratitude towards the healthcare system. Patients’ motivation to participate was hardly related to the possible benefits of GP care. In a few cases, patients mentioned that care by a GP would be closer to home, easier, and more comfortable than care by a surgeon. One patient indicated that their experiences with the surgeon influenced their (high) expectations of GP-led care:

*‘I went to visit the surgeon a few times. He was a nice person, but he had a limited idea of survivorship care. I thought it might be more convenient, more practical to go to the GP. And I have a better relationship with my GP too. I expected my GP to look further than only the wound and the physical recovery.’*

(Patient [P]4, female [F], aged 62 years)

Patients had a remarkably positive attitude towards life and their disease, resulting in little expressed needs regarding care. Characteristics such as a down-to-earth mentality, seeing the relativity of things, and always looking at things on the bright side were mentioned. Patients generally recovered quickly from their (often laparoscopic) surgery and had few symptoms. Most did not consider themselves to be a cancer patient any more:

*‘I quickly recovered from the operation, and I experienced no problems at all* […] *When you need no further treatment, you don’t feel like a patient any more. It’s done, I’m moving on* […] *I was a cancer patient for a moment, but they took the disease out, so it’s done, I am disease-free now.’*

(P2, male [M], aged 70 years)

Some patients mentioned anxiety and fear of possible cancer recurrence, especially shortly after treatment. Patients did not see an important role for their GP or surgeon when it came to psychological care, but managed these symptoms themselves, or with the support from their family and friends:

*‘You don’t really dwell on it that much. It’s more of a fact. Also the aftercare programme that was offered in the hospital. Whether I wanted to have spiritual or psychological care. No, I didn’t need that. It was done and I’m just going to go live life.’*

(P11, M, aged 71 years)

### Experiences with care

Despite the different setting, patients’ experiences with GP-led and surgeon-led care were very similar. Consultations took place by telephone or onsite. In both groups the follow-up schedule served as a guide to organise care. Patients valued the schedule, and some were able to reproduce the schedule from memory. Follow-up consultations were typically short, medically oriented, and centred around discussing the follow-up test results:

*‘I went to the doctor for the medical side. That’s all I wanted to know. Is everything OK? How is my blood test result? How are the other test results? How is the imaging result? And yes, everything else […] I am done with it. It’s over.’*

(P7, F, aged 66 years)

Patients described it as a ‘technical procedure’ or as ‘a box that needs to be checked’. Regular follow-up tests were appreciated by patients as they offered reassurance. Several patients expressed the wish to continue the follow-up testing even after completion of the follow-up schedule, assuming that their healthcare provider would accommodate:

*‘Maybe after the study period, I will continue to have my CEA value* [tumour marker] *checked once in a while. Just to keep an eye on it. Because after this 5-year period, everything stops, right? Maybe every six or twelve months I would like an additional check-up. And I assume my GP will facilitate that.’*

(P1, M, aged 48 years)

During follow-up consultations, GPs and surgeons paid attention to the general wellbeing of the patient, followed by asking some targeted questions about colon cancer or its treatment. Consultations were described according to the same structure:

*‘I entered her office. I took off my jacket and sat down. The first thing she asked me was how I was doing* […] *And then she asked her questions. These questions were if I had any complaints, especially in my abdominal area. And we discussed the fact that I gained a lot of weight. That sort of questions were asked. And that was pretty much the same every visit. A few times we spoke about my mental wellbeing and how this disease affected me.’*

(P9, M, aged 64 years)

Most patients experienced little or no symptoms, but some suffered from symptoms such as fatigue and polyneuropathy (related to adjuvant chemotherapy treatment). GPs and surgeons provided care for these symptoms, or referred the patient for specialised care. Patients who had a (temporary) stoma often received additional help by a specialised stoma nurse. Topics such as lifestyle, preventive care, and other chronic care were hardly ever discussed by GP or surgeon, though patients often did not consider it part of their physicians’ task.

The majority of patients were satisfied with the type of care they received. However, patients from both groups doubted whether GPs had sufficient knowledge to take care of patients during the whole disease trajectory. Patients receiving care by their GP sometimes noticed that the GP had difficulties interpreting test results and acting on it. Some patients would therefore prefer to contact the specialist for cancer-related problems:

*‘If I need to know something about cancer, I have to call the hospital* […] *A GP is there for the general health issues. I would call the hospital sometimes, and they gave me sufficient answers to my questions. I did not contact my GP about it.’*

(P8, M, aged 76 years)

On the other hand, some patients in the surgeon-led group had regular contacts with their GP during follow-up or were advised by the surgeon to contact the GP for (seemingly) problems unrelated to cancer:

*‘I went to see her* [GP] *a couple of times. That was actually on her request rather than mine. But that was fine with me. I was actually very happy with it.’*

(P6, F, aged 50 years)

The use of Oncokompas during survivorship care was limited. Patients with access to Oncokompas mentioned having logged in once, or only vaguely remembered it. Most patients did not believe Oncokompas would have any added value for them, since they had little complaints or symptoms to begin with.

### Roles and responsibilities

Care for patients in the GP-led group was organised in different ways. Some patients and GPs made clear agreements about their roles and responsibilities at the start of the intervention, while others mentioned it changed over time. Approaches varied from solely on the patient’s initiative to solely on the GP’s initiative, and to a shared-care model in which the initiative came from both sides. Patients also experienced difficulties with receiving care, including confusion about the follow-up schedule and having no clear point of contact. Patients therefore preferred a more guiding role from their GP during follow-up. It was feared that the follow-up tests would not be carried out as scheduled if the initiative was left to the patient. One patient explained that the GP forgot to call him for a scheduled follow-up visit, resulting in a 6-month delay in receiving test results:

*‘She was supposed to call me when it was time for a new appointment. And well, that did not happen. I patiently waited for her call, after all, I had no complaints. Until my wife encouraged me to take action, and I am thankful she did so, because there was indeed something wrong with me.’*

(P12, M, aged 81 years)

The difficulties encountered by patients in the GP-led group were in contrast to the patient experiences in the surgeon-led group. Patients in the surgeon-led group ordinarily received a letter from the hospital when it was time for a follow-up appointment. This letter stated the date and time of the follow-up test, followed by an appointment with the specialist a few days later to discuss the test results. Patients in the surgeon-led group had no remarks on the division of roles and responsibilities, as it was clear that the surgeon was in the lead. The patients sometimes mentioned that involvement of the GP would be inconvenient:

*‘I would rather go to* [the hospital location] *once or twice, than having to go to the GP to talk, then going to the hospital to do the follow-up tests, and then back to the GP to discuss the results. Then I think to myself; I’d rather keep it all in one place’*

(P7, F, aged 66 years)

*‘I think it would be easier if it* [follow-up] *stayed in the hospital. There they have their own assistants who put it in the agenda, and automatically sent a letter when I have an appointment for a blood test and a colonoscopy. Otherwise, it’s just an extra link in the chain.’*

(P5, M, aged 68 years)

## DISCUSSION

### Summary

In this mixed-methods evaluation study, patients’ experiences with GP-led versus surgeon-led colon cancer survivorship care were explored. Overall judgement and quality of care, as measured by the customised CQI, were excellent for both healthcare professionals. Before follow-up, patients had little expectations of care. Follow-up consultations for colon cancer were typically short, medically oriented, and described as a technical procedure. Topics such as psychosocial impact, lifestyle, and preventive care were less frequently discussed by either healthcare professional. Patients in the GP-led group received care in different ways, ranging from solely on the patient’s initiative to shared forms of care. This sometimes led to confusion regarding the roles and responsibilities during follow-up. These experienced difficulties in the execution of care were in contrast with patients in the surgeon-led group. These patients mentioned that the surgeon was typically in the lead.

### Strengths and limitations

A possible risk of selection bias exists inherent to the design of the I CARE study.^15^ It is plausible that patients who are generally positive about their GP and the intervention are overrepresented in the study population. The researchers have tried to reveal experiences from both sides of the spectrum by purposively selecting patients for the interviews. They gained a thorough understanding of survivorship care in real-life routine practice by interviewing patients from both the GP-led and surgeon-led group. The use of both an open narrative and semi-structured component provided a solid basis for these interviews.^20^^,^^21^ In the present study, performing the interviews at various time points may have caused recall bias. It is therefore helpful that the quantitative data were collected after 1 year. The researchers believe that this approach will have helped to strengthen the findings and illustrate the experiences that were lived and perceived over time.

Measuring the quality aspects of care was challenging. The CQI is ordinarily distributed among a larger population and had to be adapted to fit the purpose of this intervention. Nevertheless, internal consistency of CQI domains was good to excellent, and single items provided further insights. Even though there were some missing data, which can lead to non-response bias, there were no important differences between the GP-led and surgeon-led group in any of the quality aspects of care. The findings are in line with previous research,^25^ which contribute to the solidity of the conclusions. It should also be noted that a comparison is made between GPs who deliver this type of care for the first time versus surgeons who deliver this type of care on a daily basis. Confidence in GP-led care may therefore increase over time.

### Comparison with existing literature

Patients’ experiences with GP-led colon cancer survivorship care were, to the authors’ knowledge, only briefly discussed in two previous randomised trials.^25^^,^^26^ Wattchow *et al* showed no differences in patient satisfaction after 24 months,^25^ in line with the current results. This mixed-methods evaluation provides additional evidence by measuring quality aspects of care and exploring patients’ experiences in depth. In contrast to the hypothesis of the trial,^11^ greater GP involvement did not improve patients’ satisfaction or cancer care experiences. Ratings of the quality aspects of care were almost equal for both groups of healthcare professionals. This perceived equality in care could have several reasons. First, the I CARE study was a pragmatic randomised trial, in which patients and GPs were free to organise care as they thought best, but were instructed to adhere to the national guidelines for follow-up of colorectal cancer (Box 1). Care for patients with colon cancer is highly protocolised,^19^ and this may explain why consultations with both the GP and surgeon were typically short, medically oriented, and centred around follow-up test results. Second, this was a group of well-performing patients with colon cancer, who recovered quickly after surgery and experienced few symptoms,^12^ so there seemed to be little need for supportive care.

### Implications for practice

To help organise survivorship care, patients require a clear division of roles and responsibilities, resulting in adequate monitoring and surveillance of cancer. Even though patients reported few symptoms, some patients desired a more guiding role from their GP. These findings are in line with previous qualitative studies.^28^ To address all supportive care needs and help identify potential problems, physicians could use supportive care screening tools, such as the Distress Thermometer.^29^ This may also help to organise care by prioritising topics for subsequent consultations and improve confidence in care. Another way to support care coordination is through the use of a survivorship care plan, though this does not seem to affect patient outcomes.^14^ The limited use of Oncokompas was disappointing, but probably caused by the poor introduction of it to patients, where the researchers did not stimulate using this instrument (as it was part of a pragmatic trial). It might be expected that with a more stimulating introduction the usage of this instrument by patients would be more intensive.^30^

Multiple outcomes have to be taken into account when considering alternative models of care for cancer survivors, including patients’ experiences.^14^ Even though survivorship care by the GP seems possible, it is unlikely to improve patient experiences. Involvement of the GP is therefore still debatable. Other outcomes, such as costs and survival of cancer survivors, will play an important part and should be considered altogether. In order for the GP to take over care completely, further financial support and training should be provided.^27^ Of course the preferences of patients and GPs will also play an important part in this decision.

## Data

Data can be made available at the end of study, and after anonymisation, on request to the corresponding author, taking into account possible national and international legal restrictions.
